# The Oral Microbiome in Periodontal Health

**DOI:** 10.3389/fcimb.2021.629723

**Published:** 2021-03-22

**Authors:** Magdalena Lenartova, Barbora Tesinska, Tatjana Janatova, Ondrej Hrebicek, Jaroslav Mysak, Jiri Janata, Lucie Najmanova

**Affiliations:** ^1^ Institute of Microbiology, Academy of Sciences of the Czech Republic, Prague, Czechia; ^2^ Department of Genetics and Microbiology, Faculty of Science, Charles University, Prague, Czechia; ^3^ Institute of Dental Medicine, First Faculty of Medicine, Charles University and General University Hospital, Prague, Czechia; ^4^ Institute of Microbiology v. v. i., BIOCEV, Academy of Sciences of the Czech Republic, Vestec, Czechia

**Keywords:** oral microbiome, periodontal health, periodontitis, core microbiome, stomatotype, taxonomic composition, aging

## Abstract

The estimation of oral microbiome (OM) taxonomic composition in periodontally healthy individuals can often be biased because the clinically periodontally healthy subjects for evaluation can already experience dysbiosis. Usually, they are included just based on the absence of clinical signs of periodontitis. Additionally, the age of subjects is used to be higher to correspond well with tested groups of patients with chronic periodontitis, a disorder typically associated with aging. However, the dysbiosis of the OM precedes the clinical signs of the disease by many months or even years. The absence of periodontal pockets thus does not necessarily mean also good periodontal health and the obtained image of “healthy OM” can be distorted.To overcome this bias, we taxonomically characterized the OM in almost a hundred young students of dentistry with precise oral hygiene and no signs of periodontal disease. We compared the results with the OM composition of older periodontally healthy individuals and also a group of patients with severe periodontitis (aggressive periodontitis according to former classification system). The clustering analysis revealed not only two compact clearly separated clusters corresponding to each state of health, but also a group of samples forming an overlap between both well-pronounced states. Additionally, in the cluster of periodontally healthy samples, few outliers with atypical OM and two major stomatotypes could be distinguished, differing in the prevalence and relative abundance of two main bacterial genera: *Streptococcus* and *Veillonella*. We hypothesize that the two stomatotypes could represent the microbial succession from periodontal health to starting dysbiosis. The old and young periodontally healthy subjects do not cluster separately but a trend of the OM in older subjects to periodontitis is visible. Several bacterial genera were identified to be typically more abundant in older periodontally healthy subjects.

## Introduction

Periodontitis is the sixth most common disease worldwide (Frencken et al., 2017). The major forms of periodontal disease are gingivitis, chronic periodontitis (which can be the result of untreated gingivitis) and according to former classification also aggressive periodontitis, which differs from the chronic variant by faster and more extensive disease progression, lower age of patients, and obvious familial aggregation (Armitage and Cullinan, 2010; Van der Velden, 2017). Periodontal disease poses a set of inflammatory conditions affecting the tissues surrounding the teeth. It spreads from the gingiva into the deeper, supportive components of the periodontium: the gum, connective tissue, and the alveolar bone surrounding and supporting a tooth (Hernández et al., 2011), and in more severe cases it can lead to a tooth loss (Kirst et al., 2015). It is a complex infectious disease, where specific pathogenic bacteria growing in biofilms play a key role. It is thus the result of the interplay between subgingival biofilm and host immune response and is further affected by other local, environmental, and genetic factors (Griffen et al., 2012).

The oral cavity has, after the gut, the second largest and diverse microbiota harboring over 700 species of bacteria (Deo and Deshmukh, 2019). In health, the oral microbiome (OM) represents a well-balanced dynamic ecosystem that generally tends to keep within its typical values (Najmanova et al., 2021). The dysbiosis then leads to gingivitis and finally periodontitis (Hajishengallis, 2015). The microbial composition shift precedes the clinical signs of the disease (Liu et al., 2012). The relationship between specific groups of taxa and periodontitis has been thoroughly studied: The OM undoubtedly associated with severe periodontitis is characterized by the presence of the so-called “red complex” bacteria: *Porphyromonas gingivalis*, *Tannerella forsythia*, and *Treponema denticola* (Socransky et al., 1998). Other bacteria highly abundant in periodontal disease belong to the phyla Synergistetes, Firmicutes, Bacteroidetes, Chloroflexi (Griffen et al., 2012; Abusleme et al., 2013; Pérez-Chaparro et al., 2014; Kirst et al., 2015). On the other hand, only a few taxa have been unambiguously associated with periodontal health - mainly some members of genera *Actinomyces* and *Streptococcus* (Griffen et al., 2012; Abusleme et al., 2013; Kirst et al., 2015; Meuric et al., 2017). In addition, little is known about the succession of steps leading from periodontal health to disease. For many taxa, the unambiguous assignment to periodontal health or periodontitis has proven difficult because they often exhibited equal prevalence and relative abundance in both states of health. These include the highly abundant *Fusobacterium nucleatum*, *Veillonella parvula* and some members of *Streptococcus* sp., but also the less frequent *Lautropia mirabilis*, *Campylobacter gracilis*, or *Granulicatella adjacens*, (Abusleme et al., 2013; Tsai et al., 2018). The reason could be that in the studies the “periodontal health” is often characterized rather as opposite to periodontitis, i.e. the absence of clinical signs of the disease. Mainly the older cohort of healthy controls, however, could already experience the dysbiosis even though yet without clinical symptoms. Another substantial problem is posed by inconsistent criteria for the diagnosis of patients (variable depth and a number of periodontal pockets required for assignment to periodontitis group, individual experience of examining periodontologist) (Pérez-Chaparro et al., 2014; Deo and Deshmukh, 2019). Previously we tried to solve the problem by assignment of the taxa to the health state using a selection of the most diseased patients from a wider cohort, and, as the opposite a selection of healthy individuals with taxonomically most distant OM (Najmanova et al., 2021). A panel of thirty oral taxa undoubtedly associated with periodontitis corresponded well to previously published data but the list of taxa unambiguously assigned to periodontal health was surprisingly poor containing only seventeen species or so-called “combined taxa” (groups of taxa that could not be distinguished from each other by the used sequencing method). The assignment of dozens of other species frequently identified in the oral cavity thus remains questionable.

In this work, we aimed to describe a typical periodontally healthy oral microbiome and to extend the panel of oral taxa unambiguously associated with periodontal health. It is generally known, that changes in the OM composition naturally occur with aging (Feres et al., 2016; Belibasakis, 2018) which is likely related to the fact, that chronic periodontitis manifests mainly in older people (Eke et al., 2016; Feres et al., 2016; López et al., 2017). To avoid a possible age bias, we employed in our study a cohort of 91 periodontally healthy students of dentistry, i.e. young subjects (average age 23 years) having a very high standard of oral hygiene and thus with lower risk of dysbiosis (HY; healthy young). To verify the impact of age on the taxonomic OM composition, we also analyzed a group of 17 samples from periodontally healthy subjects older than 40 years (HO; healthy old), and for comparison, we also included a group of 45 patients with severe (former aggressive) periodontitis (AP). Two distinct health-associated microbial communities were identified and the dysbiotic changes that could lead to periodontitis onset were described.

## Materials and Methods

### Characteristics of Human Subjects and Sample Collection

Samples from 153 subjects were included and analyzed in this study (**Supplementary Table 1**): 91 periodontally healthy students of dentistry from the 1^st^ faculty of medicine, Charles University in Prague (average age 23 years, marked HY), 17 periodontally healthy people older than 40 years (average age 46 years, marked HO), and 45 patients with severe (former aggressive) periodontitis (average age 33, marked AP). All subjects live in Czech Republic, but besides being students of the same university in HY group, they have no other general relation among each other in terms of living area, employment or any similar parameter. All subjects were examined by a single experienced periodontologist. To be included in the HY or HO group the subjects were required to have no periodontal pocket on probing depth >3 mm, in AP group the subjects had at least two periodontal pockets on probing depth >5mm. The subjects had not received any antibiotic treatment or periodontal therapy in the three months before the beginning of the study. The HO and AP subjects were obtained within the study approved by the Ethics Committee of the First Faculty of Medicine of Charles University and General University Hospital in Prague as a part of project No. 17-30753A of the Czech Health Research Council and besides periodontitis in the AP group, they were of good general health, the sampling of HY subjects was approved within a project No. 486417 from the Grant Agency of Charles University. All subjects involved in the study signed the informed written consent.

The healthy subjects were sampled from the vestibular side of sulcus gingivalis, the samples from patients with severe periodontitis (AP) were taken from the deepest periodontal pocket. The healthy subjects fulfilled the criteria of periodontal health, as defined by Caton et al., (Caton et al., 2018), i.e. no positive bleeding on probing index (BOP), and no signs of inflammation (erythema and edema), the PPD (periodontal pocket depth) was < 2 mm, as well as the CAL (clinical attachment loss) index. The probands included in the AP group have never been treated for periodontitis prior to inclusion in our study and according to their clinical examination, their severe periodontitis diagnosis was confirmed. The clinical examination contained PPD, BOP, CAL (**Supplementary Table 1**), evaluation of the plaque and tartar amount (low for all probands), number of teeth after preservation treatment (low number of teeth with dental filling for all probands), rtg examination (prevailing vertical character of bone resorption) and family anamnesis with predominated preterm teeth loss in parents of our probands before the age of forty.

Additional information on the tooth sampled (identification of sampled tooth, CAL, BOP, PPD) and other relevant conditions including health state, smoking status, pregnancy, nationality or specific diet are listed in the **Supplementary Table 1**. The descriptive statistics on demographics and clinical data related to tested subjects is given at list “descriptive statistics” of this **Supplementary Table 1**. Each sample was obtained using two sterile paper points (BECHT, Germany). The paper points were left in the gingival sulcus or periodontal pockets for 10s to soak the fluid. Both paper points from one sampling were stored together in -20°C prior to further processing.

### DNA Isolation, 16S rDNA Gene Library Preparation, and Sequencing

The DNA was extracted using DNeasy Blood&Tissue kit (Qiagen, Germany) according to the modified manufacturer’s instructions (decreased final elution volume of AE buffer from 200 to 120 µl) and stored in -20°C. Isolated DNA was used as a template for PCR 16S rDNA amplification. The universal primers 530f (GTGCCAGCMGCNGCGG) (Dowd et al., 2008) and 907R (CCGTCAATTCMTTTGAGTTT) (Lane et al., 1985) were used to amplify the V4-V5 region of bacterial 16S rDNA in primary PCR. Primers for secondary PCR amplification contained additionally five to seven nucleotide long sample tags, separated from primers by two nucleotide long spacers (**Supplementary Table 2**). PCR reactions were performed in two steps according to Baldrian et al. (Baldrian et al., 2012). First PCR amplification was performed in 3 independent reactions for each sample in 12,5 µl Plain Combi PP Master Mix (Top-Bio, Czech Republic) containing 0,4% Phusion polymerase (New England Biolabs, USA) with 2 µl of template, 2 µl of each primer (0.25 mM) and 6,5 µl H_2_O. Cycling conditions were 94°C for 5 min; 35 cycles of 94°C for 1 min, 58°C for 50 s, 72°C for 30 s, followed by 72°C for 10 min. Pooled PCR products were purified after the electrophoretic separation from the agarose gel using the Wizard SV Gel and PCR Clean-Up System (Promega, USA). 3 µl of isolated DNA were used as a template in the second PCR in reaction containing 2 µl of each tagged primer and 25 µl of Plain Combi PP Master Mix enriched by Phusion DNA polymerase and 18 µl of H_2_O. Cycling conditions were the same except that cycle number was 10 and number of independent reactions per sample was two. PCR products were separated by electrophoresis and purified using the Wizard SV Gel and PCR Clean-Up System and then concentrated into the volume of 16 µl using the MinElute PCR Purification Kit (QIAGEN, Germany). The concentration of DNA was measured by Qubit 2.0 Fluorometer using dsDNA BR Assay Kit (both Thermo Fisher Scientific, USA). The purified solutions of tagged amplicons from different samples were mixed in equimolar concentrations, the amplicon library was constructed using TruSeq DNA Library Preparation Kit v2 (Illumina, USA) and sequenced by Illumina MiSeq platform (paired-end reads, 2×250 bp).

### Analysis of the Sequencing Data

The amplicon raw sequencing data were processed using the pipeline SEED 2.0.3 (Větrovský et al., 2018). The pair-end reads were joined using fastq-join program as a part of ea-utils package (Aronesty, 2013). All sequences were trimmed to 365 nt starting from the first nucleotide after the end of the forward primer sequence. The trimmed sequences were further clustered to 98.5% sequence identity by USEARCH (Edgar, 2010) implemented in SEED and the chimeric sequences were removed (chimera check is a part of the clustering method using Uparse algorithm). The quality-filtration to the mean quality Phred score treshold 30 was applied. Consensus sequences were constructed for each cluster and then compared to HOMD database (Chen et al., 2010) using the Blastn tool. Because not the whole length, but only a 365 nt long portion of the 16S rDNA sequence was analyzed, more human oral taxa (HMTs) often cluster to each consensus at the 98.5% level of identity resulting in the ambiguous taxonomic assignment. To overcome this problem, we defined the “combined taxa” (CTs) in cases when the precise assignment to a single HMT was not possible. The CTs were defined as follows: 889 sequences from HOMD version 14.51 were trimmed to 365 nt equally to our testing sequences and clustered at 98.5% identity resulting in 293 single HMTs and 106 clusters containing from 2 to 15 HMTs. These clusters were named CT1 – CT106 (**Supplementary Table 3**). The identified numbers of reads per each taxon were further normalized to taxon relative abundance value with respect to a 16S rDNA copy number per genome. This procedure was demonstrated to result in a more realistic estimate of the relative abundances of the bacterial taxa, as it takes into account the variation of 16S rDNA copy numbers among taxa (ranging from 1 to 15). The bacterial genome count estimates were calculated based on the 16S rDNA copy numbers in the closest available sequenced genome as described previously by Vetrovsky and Baldrian (Větrovský and Baldrian, 2013). For the purposes of this publication, the published table of the 16S rDNA copy numbers was extended using rrnDB database (Stoddard et al., 2015) (**Supplementary Table 4**). The sequencing statistics including diversity indices and rarefaction curves is summarized in **Supplementary Table 7**). The raw sequences are deposited in the NCBI Short Read Archive (BioProject accession no. PRJNA670573).

### Bioinformatic Analysis

**Figure 1:**
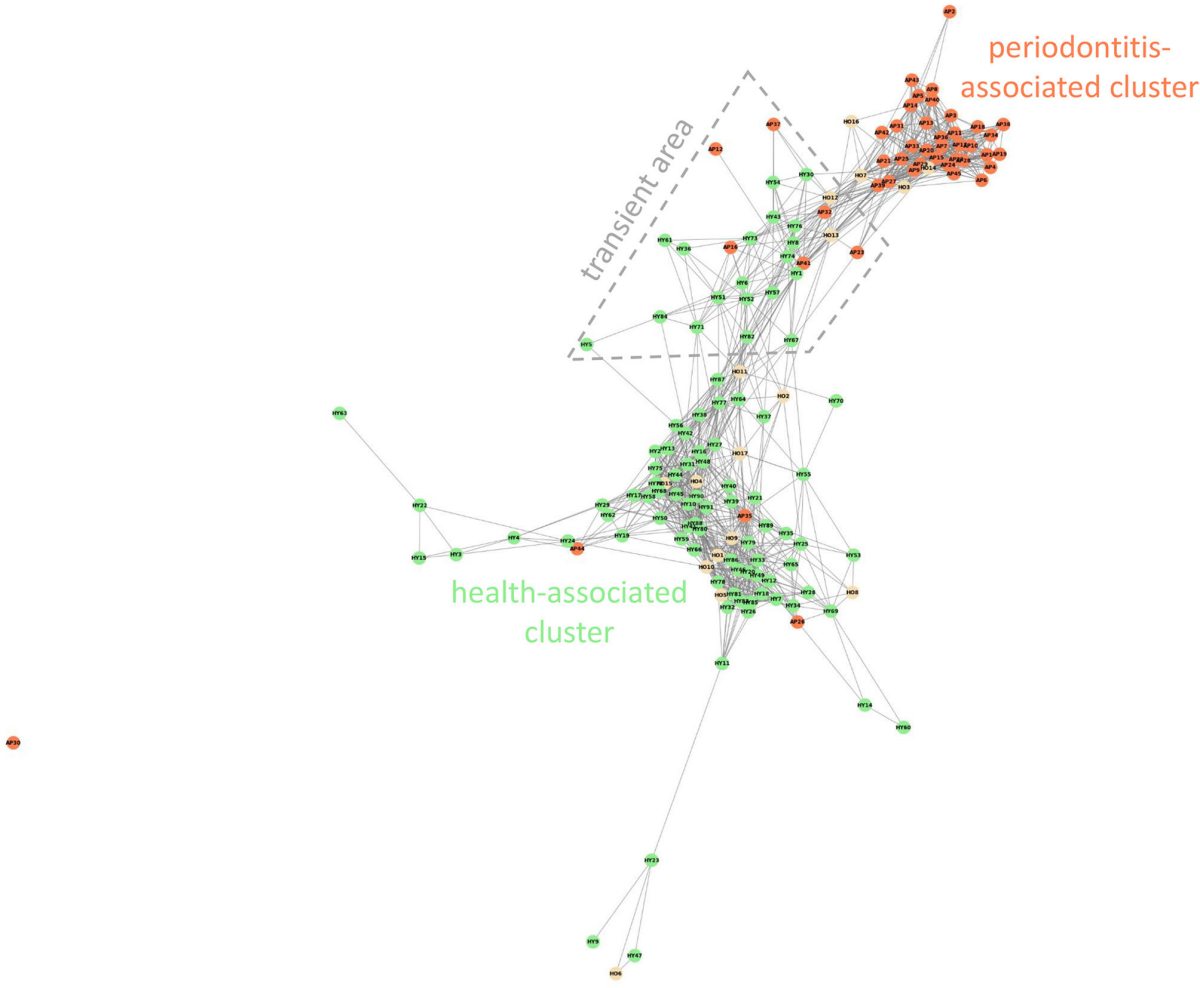
Weighted Jaccard Similarity clustering analysis of all OM samples. Weighted Jaccard similarity index 0.3. The red-colored spots correspond to patients with AP, HY samples are green and HO samples are yellow. The transient area is delimited with the gray dashed line.

The Weighted Jaccard Similarity clustering analysis (**Figure 1**) was performed using the Python programming language (Rossum and Drake, 2010), specifically the NetworkX library (Hagberg et al., 2008). The network graph was generated based on **Supplementary Table 5**, which displays the relative per-sample abundances of all taxa, with abundances below 0.05% rounded down to zero as insignificant. To reduce the dimensionality of the data, a similarity matrix was calculated between all samples using an abundance-weighted Jaccard similarity index. This highlighted the similarities in the overall composition of the biofilm, rather than taxonomic diversity. In other words, the impact of low-abundance taxa on the similarity metric was reduced. For visualization, the aforementioned NetworkX library was used to create a network graph. The generated graph is unweighted, meaning a shorter distance between nodes does not always reflect greater similarity. Links were displayed only between nodes with a similarity index of 0.3 or greater.

The PCA (**Figure 3**) and multivariate clustering analysis (**Figure 4**) were performed in the free statistical software PAST 3.25 (Hammer et al., 2001) using the processed sequencing data summarized in **Supplementary Table 5**. The paired group (UPGMA) algorithm and the Bray-Curtis similarity index were employed to obtain **Figure 4**. The statistical significance of identified group characteristics was further analyzed using one-way PERMANOVA test of CLR (central log ratio) transformed data in PAST 3.25. A SIMPER (Similarity Percentage) test within PAST 3.25 software was used for assessing which taxa are primarily responsible for observed differences between groups of samples and One-way ANOVA with Dunn´s *post hoc* test was employed to assess the statistical significance of individual taxa relative abundance difference among groups identified by multivariate clustering analysis (**Supplementary Table 6**).

## Results

The sequencing statistics is summarized in the **Supplementary Table 7**. In average we obtained 11334 reads/sample for HY group, 16709 for HO group, and 11974 for AP group with minimum 1006 reads/sample. The rarefaction curves were calculated for a random selection of 1000 sequences to reflect the minimum sample size, set up to cover the expected relevant taxonomic diversity of oral microbiome samples (the sequencing was repeated when the number of reeds per sample was < 1000 to ensure identification of all taxa exceeding average relative abundance 0.5%). The diversity, evenness, and species richness parameters were estimated for each sample (**Supplementary Table 7**) and were compared also between HY HO and AP groups using one-way ANOVA with Dunn´s *post hoc* test. No statistically significant difference was identified for any parameter except for the Chao-1 richness, statistically significantly higher in HY group when compared to AP (p = 0.002); for HY/HO and HO/HP p was > 0.05.

### Healthy and Diseased Samples Cluster Mostly Separately but Few of Them Are Always Misclassified

All 153 samples were clustered using Weighted Jaccard Similarity analysis, index value 0.3 (**Figure 1**). For each sample, all taxa with relative abundance value >0.05% were included in the analysis. Two major distinct clusters were observed: the periodontitis-associated cluster containing mainly samples of AP patients (red spots in **Figure 1**) and the health-associated cluster covering mainly subjects without clinical signs of the disease (green and yellow spots in **Figure 1**) with several samples from all three cohorts localized in the area in between. The periodontitis-associated cluster contains 39 samples (35 AP and 4 HO), the health-associated cluster includes 86 samples (72 HY, 11 HO, and 3 AP). Twenty-seven samples are localized in the area connecting both the above-mentioned clusters (transient area, dashed line in **Figure 1**) and one sample (AP30) was an outlier, not included in further analysis. The transient area contains 6 AP and 21 H samples (19 HY + 2 HO). The one-way PERMANOVA test confirmed the difference among the healthy, periodontitis and transient groups with p=0.0001 for all mutual comparisons.

In the health-associated cluster, the most abundant and prevalent taxa are CT2 Streptococcus mitis/S. oralis, CT43 Streptococcus gordonii/S. sanguinis, CT6 Veillonella dispar/V. parvula, CT25 Neisseria flava/N. mucosa, CT27 Neisseria subflava, HMT14 Neisseria oralis, HMT718 Haemophilus parainfluenzae, CT23 Haemophilus haemolyticus, CT24 Haemophilus sputorum, CT10 Prevotella histicola, CT37 Gemella morbillorum, CT48 Rothia dentocariosa, HMT22 Lautropia mirabilis, HMT37 Stenotrophomonas nitritireducens, and CT13 Aggregatibacter aphrophilus (**Table 1**). The relative abundance and in most cases also the prevalence of these taxa remarkably decreases toward the transient area and periodontitis-associated cluster. On the other hand, the relative abundance and prevalence of taxa typical for periodontitis do not reach high values in periodontal health and transient area, but the increasing trend from the health, through the transient area to the periodontitis-associated cluster is noticeable. Several taxa exhibit the highest abundance and prevalence in subjects from the transient area, namely CT3 *Fusobacterium nucleatum*, CT8 *Porphyromonas pasteri*/*P. catoniae*, HMT775 *Capnocytophaga sputigena*, HMT329 *Capnocytophaga leadbetteri* and CT51 *Capnocytophaga granulosa*, HMT311 *Prevotella oris*, CT53 *Tannerella* sp. and HMT623 *Campylobacter gracilis.*

**Table 1:** The average relative abundance and representation of selected taxa in the health-associated cluster, transient area, and periodontitis-associated cluster.

HMT/CT	*health-associated cluster*		*transient area*		*periodontitis-associated cluster*	
	average abundance [%]	prevalence [%]	average abundance [%]	prevalence [%]	average abundance [%]	prevalence [%]
CT2 *Streptococcus mitis; S. oralis*	20.87	100.00	4.37	96.30	0.52	48.72
CT6 *Veillonella dispar; V. parvula*	15.19	100.00	5.15	100.00	0.51	53.85
CT25 *Neisseria flava; N. mucosa*	6.54	88.37	2.37	74.07	0.89	28.21
CT43 *Streptococcus gordonii; S. sanguinis*	4.97	100.00	0.65	74.07	0.12	33.33
HMT718 *Haemophilus parainfluenzae*	4.83	96.51	0.60	70.37	0.05	20.51
CT10 *Prevotella histicola*	2.48	84.88	0.71	77.78	0.17	38.46
CT27 *Neisseria subflava*	2.45	87.21	0.61	77.78	0.24	38.46
HMT14 *Neisseria oralis*	2.43	53.49	1.50	62.96	0.12	23.08
CT37 *Gemella morbillorum*	2.18	89.53	1.09	85.19	0.10	33.33
CT48 *Rothia dentocariosa*	1.95	74.42	0.31	37.04	0.17	17.95
HMT22 *Lautropia mirabilis*	1.93	80.23	0.40	62.96	0.09	25.64
CT23 *Haemophilus haemolyticus*	1.44	80.23	0.27	62.96	0.02	5.13
HMT37 *Stenotrophomonas nitritireducens*	1.13	69.77	0.05	22.22	0.09	10.26
CT24 *Haemophilus sputorum*	1.07	66.28	0.17	44.44	0.00	5.13
CT13 *Aggregatibacter aphrophilus*	1.00	56.98	0.43	59.26	0.14	17.95
CT3 *Fusobacterium nucleatum*	4.76	97.67	31.30	100.00	23.32	100.00
CT8 *Porphyromonas pasteri; P. catoniae*	2.15	82.56	13.05	92.59	0.37	41.03
HMT775 *Capnocytophaga sputigena*	1.37	63.95	2.63	77.78	0.09	23.08
HMT329 *Capnocytophaga leadbetteri*	0.56	44.19	1.91	62.96	0.12	25.64
HMT311 *Prevotella oris*	0.26	34.88	1.20	55.56	0.68	20.51
CT51 *Capnocytophaga granulosa*	0.97	34.88	1.15	66.67	0.32	43.59
CT53 *Tannerella* sp.	0.23	32.56	1.06	59.26	0.23	33.33
HMT623 *Campylobacter gracilis*	0.38	43.02	1.02	88.89	0.35	64.10
HMT619 *Porphyromonas gingivalis*	0.02	6.98	0.13	14.81	12.15	74.36
HMT613 *Tannerella forsythia*	0.01	5.81	0.21	25.93	7.29	100.00
CT12 *Fretibacterium* sp.	0.00	2.33	0.13	37.04	6.55	97.44
CT50 *Treponema denticola*	0.02	8.14	0.30	25.93	5.91	94.87
CT22 *Porphyromonas endodontalis*	0.20	13.95	1.34	37.04	4.17	94.87
CT7 *Treponema vincentii*	0.02	9.30	0.78	44.44	3.59	92.31
CT11 *Treponema socranskii*	0.04	13.95	0.90	48.15	3.25	100.00
HMT643 *Prevotella intermedia*	0.20	5.81	2.04	25.93	2.70	74.36
HMT274 *Bacteroidales [G-2]* sp.	0.13	11.63	0.29	37.04	2.38	69.23
HMT539 *Filifactor alocis*	0.05	6.98	0.03	18.52	1.57	89.74
CT56 *Campylobacter rectus*	0.17	36.05	0.99	70.37	1.49	87.18
HMT363 *Fretibacterium fastidiosum*	0.01	2.33	0.04	14.81	1.46	97.44
CT42 *Treponema maltophilum*	0.00	3.49	0.05	18.52	1.17	94.87

The oral taxa predominantly abundant and prevalent in the health-associated cluster are marked green. Taxa with the highest abundance and representation in the transient area are gray and the taxa with the highest average relative abundance and prevalence in the periodontitis-associated cluster are red. The table includes only the taxa with minimal average relative abundance >1% and minimal prevalence >50% in at least one cluster. CT stands for combined taxon (See **Supplementary Table 3**).

Even though no obvious age-dependent clustering pattern was observed, still 35% of samples from HO group (6 out of 17), clustered together with AP or in close proximity (yellow spots in **Figure 1**), but no HY sample clustered together with AP. We compared the OM taxonomic composition of these 6 HO samples (HO3, 7, 12-14, and 16; highlighted in **Supplementary Table 5**) with a group of remaining 102 samples from healthy individuals (HY and HO) and also a group of 45 AP samples. The OM of these 6 HO samples resembles the diseased one, just the relative abundance and prevalence values of main periodontitis-associated taxa (red-complex taxa, *Fretibacteria* CT12, and HMT 363, and *Filifactor alocis* HMT 539) are slightly lower when compared to the AP group. On the other hand, the average relative abundance of *Fusobacterium nucleatum* is higher in these six samples. The taxa typical for periodontal health (CT2 *Streptococcus mitis/S. oralis*, CT6 *Veillonella dispar/V. parvula*, CT25 *Neisseria flava/N. mucosa*) are almost absent exhibiting the average relative abundance values comparable to the AP group. Even though the 6 HO individuals did not exhibit any clinical signs of the disease, their OM taxonomic composition indicates a high risk of periodontitis development in the future.

### The OM Taxonomical Composition in Aging

**Figure 2:**
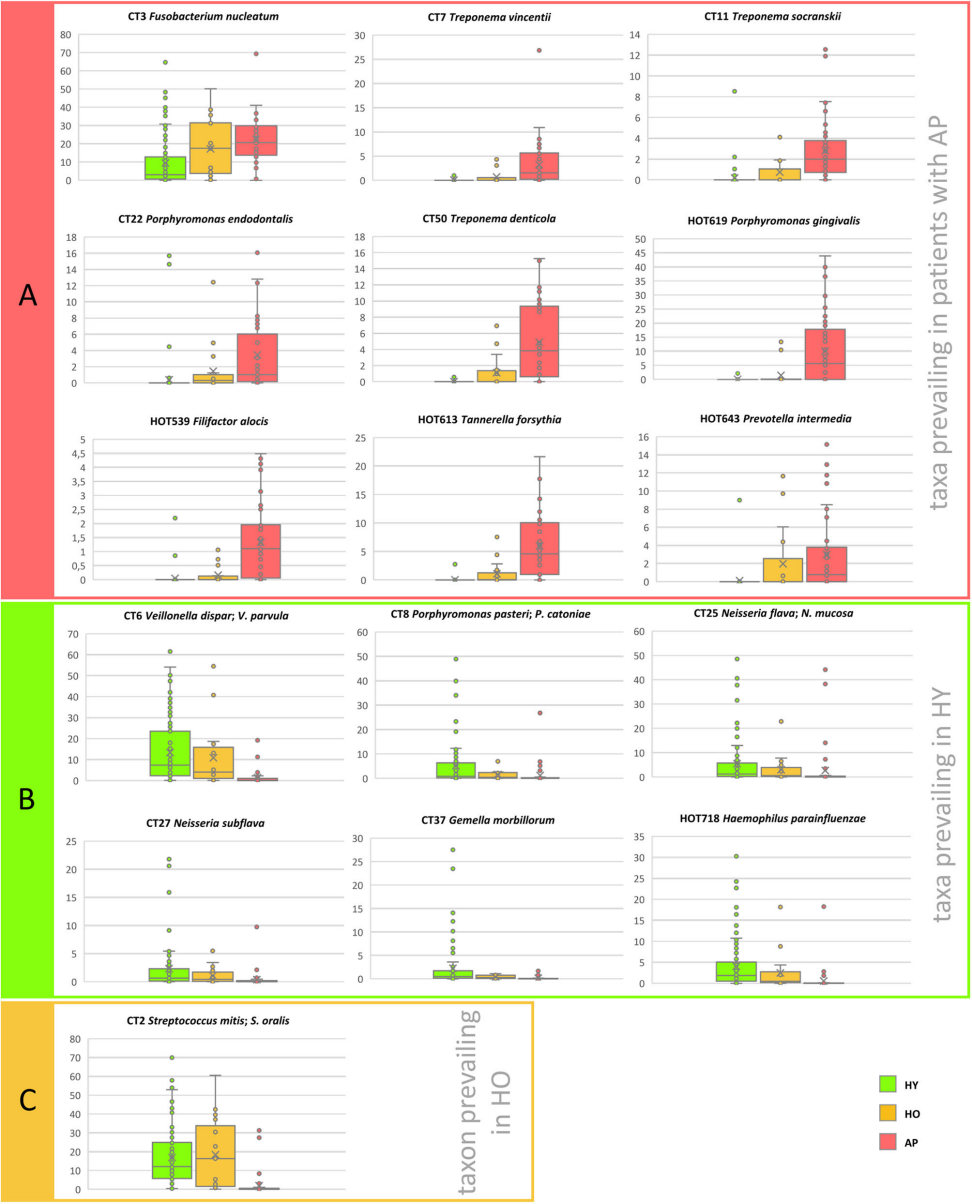
The distribution of the relative abundances of selected taxa in relation to the age of probands and their state of health. **(A)** Taxa prevailing in periodontitis, **(B)** Taxa prevailing in HY, **(C)** Taxon prevailing in HO. The area of box plots with oral taxa dominant in patients with AP is highlighted in red, HY in green and one boxplot related to taxon most abundant in the HO group is highlighted in yellow. CT stands for combined taxon (See **Supplementary Table 3**).

The relative abundance values of selected taxa in relation to the age and state of health are compared in **Figure 2** and summarized including the prevalence data in **Table 2**. Three groups of samples were compared: HY (average age 23), HO (average age 46), and patients with AP (average age 33). The one-way PERMANOVA test revealed clear difference between groups. When Bray-Curtis was used as a similarity index, the HY and HO were found close to each other (0.0059) but clearly different from AP (0.94 and 0.86, resp.). Twelve taxa were identified to be the most abundant in HY: CT6 *Veillonella dispar*/*V. parvula*, CT8 *Porphyromonas pasteri*; *P. catoniae*, CT25 *Neisseria flava*/*N. mucosa*, HMT718 *Haemophilus parainfluenzae*, CT43 *Streptococcus gordonii*/*S. sanguinis*, CT10 *Prevotella histicola*, CT27 *Neisseria subflava*, CT37 *Gemella morbillorum*, HMT14 *Neisseria oralis*, HMT22 *Lautropia mirabilis*, CT48 *Rothia dentocariosa*, and CT23 *Haemophilus haemolyticus*. A nicely visible gradual trend of diminution can be seen in the abundance and in most cases also in prevalence of these taxa toward to group of HO and patients with AP. On the other hand, in the AP group the highest abundance of periodontitis-associated taxa CT3 *Fusobacterium nucleatum*, CT7 *Treponema vincentii*, CT11 *Treponema socranskii*, CT50 *Treponema denticola*, CT12 *Fretibacterium* sp., CT22 *Porphyromonas endodontalis*, HMT619 *Porphyromonas gingivalis*, CT56 *Campylobacter rectus*, HMT274 *Bacteroidales [G-2]* sp., HMT363 *Fretibacterium fastidiosum*, HMT539 *Filifactor alocis*, HMT613 *Tannerella forsythia*, and HMT643 *Prevotella intermedia* was identified with an opposite trend of subsequent decreasing of the abundance and prevalence of these taxa toward to HO and then to HY subjects. Only a few bacterial taxa were found to prevail in the group of HO samples: CT2 *Streptococcus mitis*/*S. oralis*, CT51 *Capnocytophaga granulosa*, HMT329 *Capnocytophaga leadbetteri*, HMT775 *Capnocytophaga sputigena*, and HMT322 *Bergeyella* sp.

**Table 2:** The comparison of the relative abundance and prevalence of significant oral taxa according to the age (HY vs HO) and state of health (HY, HO and AP).

HMT/CT	*HY*		*HO*		*AP*	
	average abundance [%]	prevalence[%]	average abundance [%]	prevalence[%]	average abundance [%]	prevalence[%]
CT6 *Veillonella dispar; V. parvula*	13.37	100.00	10.87	100.00	1.46	60.00
CT8 *Porphyromonas pasteri; P. catoniae*	5.19	85.71	1.59	88.24	1.19	44.44
CT25 *Neisseria flava; N. mucosa*	5.44	83.52	3.03	94.12	2.66	35.56
HMT718 *Haemophilus parainfluenzae*	4.02	92.31	2.47	88.24	0.56	26.67
CT43 *Streptococcus gordonii; S. sanguinis*	3.98	96.70	2.35	82.35	1.27	40.00
CT10 *Prevotella histicola*	2.37	84.62	0.77	82.35	0.25	42.22
CT27 *Neisseria subflava*	2.31	86.81	1.09	76.47	0.38	44.44
CT37 *Gemella morbillorum*	2.27	89.01	0.43	94.12	0.16	37.78
HMT14 *Neisseria oralis*	2.17	54.95	0.77	64.71	1.09	26.67
HMT22 *Lautropia mirabilis*	1.82	82.42	0.23	47.06	0.25	31.11
CT48 *Rothia dentocariosa*	1.64	68.13	1.05	52.94	0.43	24.44
CT23 *Haemophilus haemolyticus*	1.37	81.32	0.38	64.71	0.01	6.67
CT2 *Streptococcus mitis; S. oralis*	16.94	100.00	18.38	100.00	1.95	53.33
CT51 *Capnocytophaga granulosa*	1.04	40.66	1.24	58.82	0.24	20.00
HMT322 *Bergeyella* sp.	0.60	70.33	1.73	70.59	0.04	13.33
HMT329 *Capnocytophaga leadbetteri*	0.68	48.35	1.63	58.82	0.32	24.44
HMT775 *Capnocytophaga sputigena*	1.74	64.84	1.39	82.35	0.23	26.67
CT3 *Fusobacterium nucleatum*	9.48	98.90	17.23	100.00	22.44	97.78
CT7 *Treponema vincentii*	0.05	12.09	0.71	35.29	3.25	88.89
CT11 *Treponema socranskii*	0.17	17.58	0.75	47.06	2.80	88.89
CT12 *Fretibacterium* sp.	0.01	5.49	1.03	35.29	5.36	88.89
CT22 *Porphyromonas endodontalis*	0.40	9.89	1.45	58.82	3.46	91.11
CT50 *Treponema denticola*	0.02	4.40	1.11	47.06	4.90	86.67
CT56 *Campylobacter rectus*	0.38	40.66	0.63	64.71	1.21	82.22
HMT274 *Bacteroidales [G-2]* sp.	0.16	13.19	0.98	47.06	1.79	60.00
HMT363 *Fretibacterium fastidiosum*	0.01	3.30	0.38	35.29	1.14	77.78
HMT539 *Filifactor alocis*	0.05	5.49	0.16	41.18	1.32	75.56
HMT613 *Tannerella forsythia*	0.04	3.30	1.07	52.94	5.99	88.89
HMT619 *Porphyromonas gingivalis*	0.03	3.30	1.44	29.41	10.04	68.89
HMT643 *Prevotella intermedia*	0.10	2.20	1.97	47.06	3.00	71.11

The taxa prevailing in each group are highlighted in respective color: green for HY, peach for HO, and red for AP. The table includes only taxa with minimal average relative abundance >1% and minimal prevalence >50% in at least one group. CT stands for combined taxon (See **Supplementary Table 3**).

### The Stomatotypes in Oral Health and Disease

**Figure 3:**
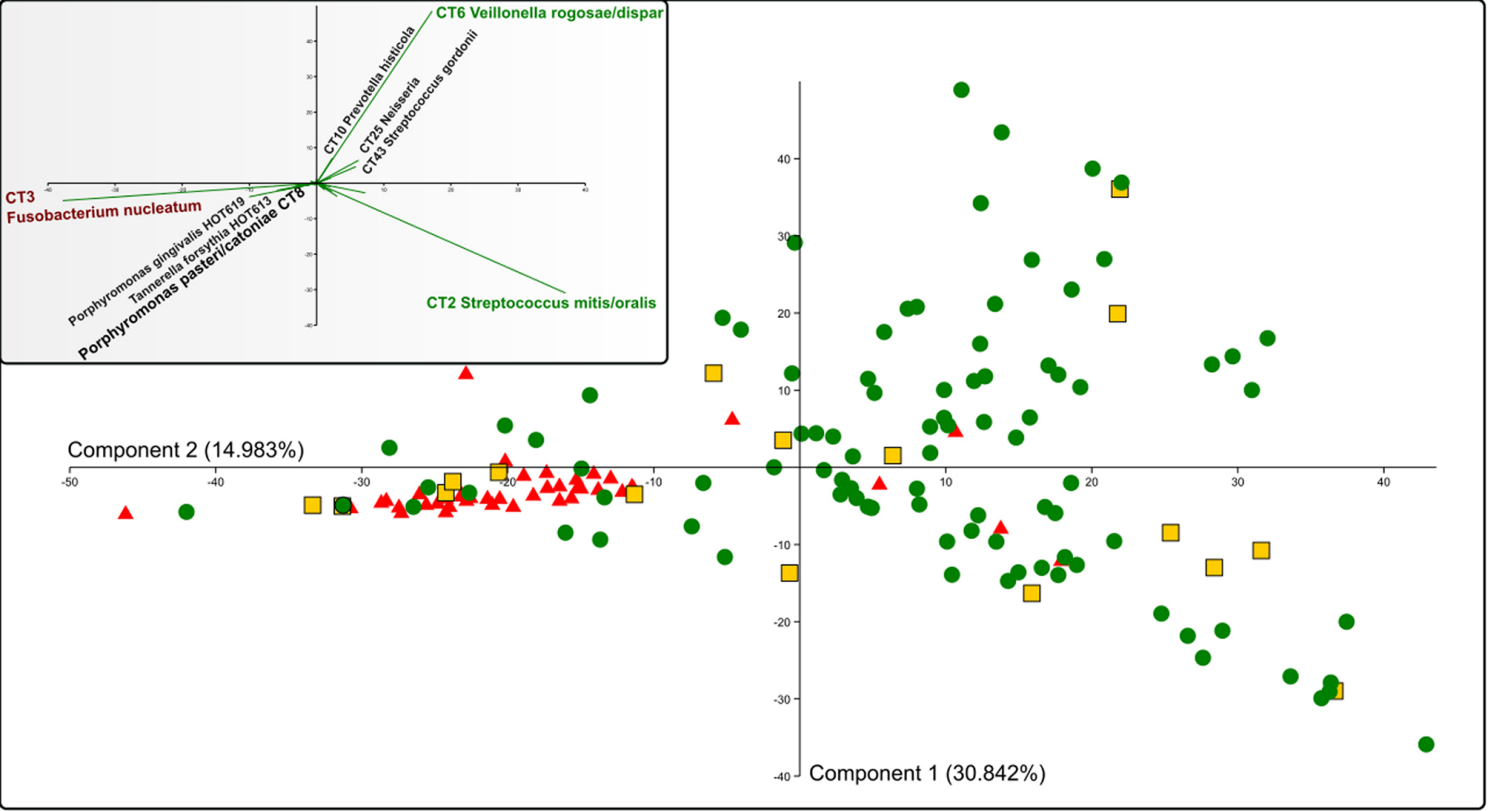
PCA analysis of all OM samples. Red triangles represent AP samples, green spots HY, and yellow squares represent the HO samples. The inner panel shows the contribution of selected oral taxa to the distribution. The determining taxa for each quadrant are highlighted in colors (green for periodontal health and red for periodontitis), CT8 P. pasteri/catoniae determining the transient state is highlighted by a bigger font. CT stands for combined taxon (See **Supplementary Table 3**).

**Figure 4:**
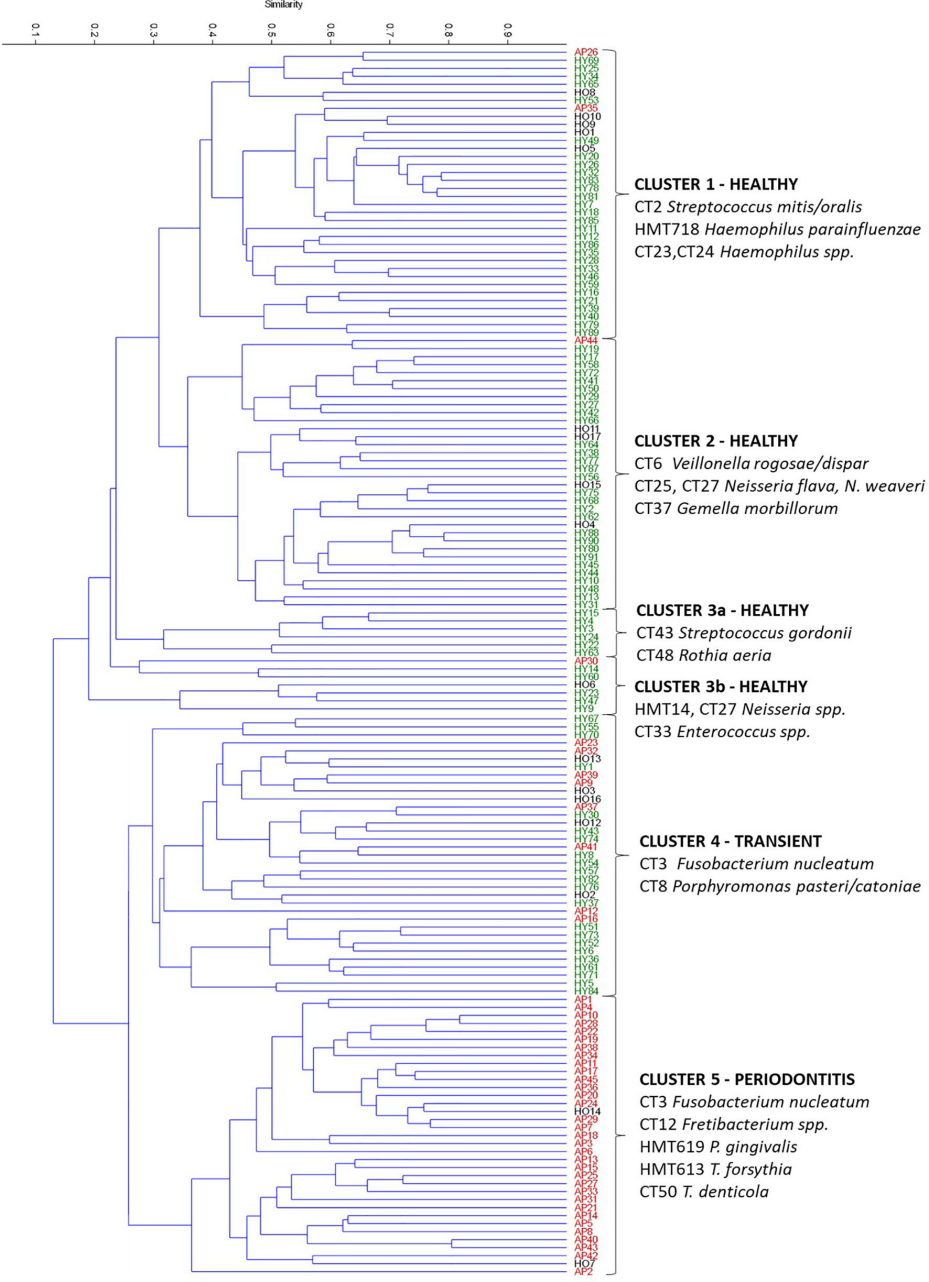
Hierarchical clustering of all OM samples based on the Bray-Curtis similarity indexes. AP samples are marked red, HO black, and HY green. The upper measure indicates the Bray-curtis value. The determining taxa for each cluster are listed. CT stands for combined taxon (See **Supplementary Table 3**).

All 153 samples were analyzed using principal component analysis (PCA; **Figure 3**) and hierarchical clustering analysis based on the Bray-Curtis similarity indexes (**Figure 4**). All identified taxa in each sample were taken into account in the calculation of sample distance. In addition, in this case, the majority of AP samples form a compact cluster (red triangles in **Figure 3**) apart from a much more diffuse group of health-associated spots (green dots and yellow squares in **Figure 3**). The inner panel in **Figure 3** shows the contribution of individual taxa to the distribution of samples.

The clustering analysis based on Bray-Curtis similarity indexes revealed two superclusters (**Figure 4**), one associated mainly with periodontal health and the second with periodontitis. The health-associated supercluster comprises two main clusters of equal size (Cluster 1 and Cluster 2) and two small clusters (Cluster 3a and 3b) corresponding to outliers from **Figure 1**. The periodontitis-associated supercluster comprises also two main clusters: Cluster 4 corresponding to the transient state, and Cluster 5 corresponding to periodontitis (compare to **Figure 1**). Using one-way PERMANOVA test, the Clusters 3a and 3b were not found to differ significantly from each other (p = 0.33), however, when considering the Clusters 1 and 2 (health-associated), 4 (transient) and 5 (periodontits-associated), they all mutually differ significantly (p = 0.0001). The determining taxa for each cluster are listed in **Figure 4** and in more detail in **Supplementary Table 6**. For twenty most discriminating taxa according to Simper test also One-way ANOVA with Dunn´s *post hoc* test was employed to compare the clusters (**Supplementary Table 6**; individual lists; statistically significant results are highlighted in red).

Predictably, the periodontitis associated cluster Cluster 5 is characterized by the high relative abundance of CT3 *F. nucleatum*, CT12 *Fretibacterium* spp., and red complex taxa HMT619 *P. gingivalis*, HMT613 *T. forsythia*, and CT50 *T. denticola*. Also, the transient state-associated cluster Cluster 4 is characterized by the high relative abundance of CT3 *F. nucleatum*, but in this case, accompanied by CT8 *Porphyromonas pasteri/catoniae* and no red-complex taxa. Two main health-associated clusters are Cluster 1 (characterized by CT2 *Streptococcus mitis/oralis* and *Haemophilus* spp. HMT718, CT23, and CT24) and Cluster 2 (characterized by CT6 *Veillonella rogosae/dispar*, *Neisseria* spp. CT25, CT27, and CT37 *Gemella morbillorum*). This distribution corresponds well also with the main taxa driving the PCA distribution (the inner panel in **Figure 3**). In contrast to the periodontitis-associated cluster, the two health-associated stomatotypes are not conclusively distinguished. They rather form a diffuse group covering all possible combinations of health-associated taxa.

## Discussion

### Misclassification of Samples

The Weighted Jaccard Similarity analysis (**Figure 1**), as well as the PCA analysis (**Figure 3**), expectedly distinguished a compact cluster of AP samples from a bigger and more diffuse cluster of samples of healthy individuals. A similar trend was observed earlier (Kirst et al., 2015) and it was explained as a result of microbial succession during the onset of periodontal disease. Kirst et al. revealed that shallow sampling sites in patients with chronic periodontitis exhibited the highest species richness and diversity (containing both, the health-associated taxa as well as the taxa typical for periodontitis), while the deep periodontal pockets contained only a limited number of species that were, moreover, quite uniform among the tested individuals. The healthy sites were also less diverse, but the individuals differed more, which could result from an ambiguous diagnosis of some healthy probands, in whom the early stages of dysbiosis can occur without any clinical signs. In our study, in order to characterize a periodontally healthy oral microbiome, we included the HY group, selected on purpose from young people with precise oral hygiene, i.e. with a very low probability of dysbiosis that could otherwise bias the results. To contrast with the former, we also included a group of severe periodontitis (AP) patients who are typically characterized by unambiguous diagnosis, deep periodontal pockets, and a rapid progression of the disease. Nevertheless, even with such a well-distinguished set of individuals, still, several HY samples cluster close to the AP group. From the HO group, in which the dysbiosis preceding the periodontitis development could already be expected, four samples cluster directly within the AP group (**Figure 1**), and few AP samples, on the other hand, cluster together with the healthy ones. The PCA analysis shows an even bigger overlap (**Figure 3**).

A similar discrepancy between the microbial profile and clinical status in some percentage of samples was already shown previously (Kirst et al., 2015; Park et al., 2015; Szafranski et al., 2015). PCA or PCoA analysis frequently revealed a compact cluster of samples from periodontitis patients, a bigger and more diffuse cluster of samples from periodontally healthy individuals, and several outliers or samples assigned to an improper group. In our study, ~18% of samples did not belong to any of the identified AP or healthy cluster but form a connection between them (**Figure 1**). We suppose that these samples represent a transient state. The clinically healthy subjects (HY and HO) from the transient area and the four HO samples clustering with AP would thus probably experience dysbiosis and consequently would be at a higher risk of periodontitis onset, while the AP samples from the transient area could correspond to a milder course of the disease or patients with better prognosis. Nevertheless, we must consider also the possibility of altered functional activity of the OM as discussed for example by Duran-Pinedo (Duran-Pinedo and Frias-Lopez, 2015) and/or an unusual host immune response, more tolerogenic in case of healthy subjects with unhealthy OM and more proinflammatory in AP subjects with transient OM (Sultan et al., 2018).

Four AP samples from our set were misclassified: One outlier (AP30), and three samples localized within the health-associated cluster in **Figure 1** (AP26, AP35, and AP44). The outlier AP30 exhibited very high relative abundance (44%) of CT98 *Propionibacterium propionicum* (**Supplementary Table 5**; list AP). The high relative abundance of *P. propionicum* is by some authors correlated with apical periodontitis and endodontal lesions, however, this finding has not been corroborated by others, and there is no consensus concerning the role of *P. propionicum* in the pathogenesis of the periodontal disease (Dioguardi et al., 2020). The three remaining samples represented typical health-associated OMs and similarly to the AP30 they did not contain any of the above-mentioned “true periopathogens”. Some authors explain this phenomenon with other causes of periodontal pocket formation then periodontitis, for example, anatomical abnormalities in labial frena (Monnet-Corti et al., 2018). This, however, is not the case of our patients. Two of them (AP26 and AP35) suffer from a localized form of the disease with 5 affected teeth, and two other (AP30 and AP44) have generalized AP with even 25 and 20 periodontal pockets, respectively. Such an extent of the disease cannot be caused by labial frena abnormalities. The severe periodontitis in these individuals thus probably originated from an unusual interplay between their OM and immune system. It is important to note, that the 16S rDNA sequencing-based taxonomic characterization of the OM provides a valuable, but still incomplete picture of the oral ecosystem. The bacteria are major and very important members of the OM, but fungi and archaea species also can play their role, and additionally, the metabolic activity of individual species can differ in relation to interactions with their surroundings (Sultan et al., 2018). Consequently, more complex diagnostic tools including proteomic or metabolomic studies will be required to reveal the cause of inflammation and periodontitis development in these nonstandard cases.

The most abundant and prevalent taxa in health and periodontitis-associated cluster are consistent with previously published data (Griffen et al., 2012; Abusleme et al., 2013; Pérez-Chaparro et al., 2014; Kirst et al., 2015). Slightly surprising could be very low prevalence of *Aggregatibacter actinomycetemcomitans* in our AP group (the taxon was identified only in three samples; 0.11% in AP24, 3.6% in AP12, and 15.8% in AP37), because for a long time, this taxon has been typically associated with periodontitis, mainly with its severe (aggressive, according to former classification) form (Schacher et al., 2007; Henderson et al., 2010). Nevertheless, Henderson et al., also document, that the proportion of the population that harbors *A. actinomycetemcomitans* varies dramatically between various geographical areas and different clinical presentations of periodontitis. For example within Europe, 23% of Dutch subjects harbored *A. actinomycetemcomitans* compared to only 3% of Spanish subjects, on the other hand in Asia it was detected in 78% of healthy Vietnamese subjects. There is no general study concerning the prevalence of *A. actinomycetemcomitans* in periodontitis patients in the Czech Republic, however, with respect to the published geographical and ethnical variability, the low *A. actinomycetemcomitans* prevalence in our cohort does not put our diagnosis of severe (aggressive) periodontitis in question.

The OM of individuals in the transient area is characterized by the decreased relative abundance of typical health-associated taxa and increased relative abundance of anaerobic or facultative anaerobic taxa (genera *Fusobacterium, Porphyromonas* or *Capnocytophaga*), which are supposed to act as later colonizers, facilitating further colonization by “true periopathogens” of red complex (Socransky et al., 1998), and/or species of periodontitis-associated genera like *Treponema*, *Fretibacterium*, or *Filifactor*. The role of *F. nucleatum* in the subgingival biofilm formation probably lies in the bridging among microorganisms, allowing attachment of periodontitis-specific bacteria (Kolenbrander et al., 2006; Kolenbrander et al., 2010). The presence of *F. nucleatum* on its own does not cause periodontal disease, however, its increased abundance is undoubtedly associated with the disease (He et al., 2012; Yang et al., 2014). The second most abundant bacteria in samples from the transient area is CT8 *Porphyromonas pasteri*/*catoniae* (13.05%). The genus *Porphyromonas* is quite unique because some *Porphyromonas* species are frequently associated with oral health (De Lillo et al., 2004; Camelo-Castillo et al., 2015; Takeshita et al., 2016; Yasunaga et al., 2017; Rusthen et al., 2019) while another member of the genus, *Porphyromonas gingivalis*, belongs to the red-complex and it is unequivocally disease-associated. Our results show that representatives of CT8 *P. pasteri* and/or *P. catoniae* do not form the core microbiome in oral health but rather indicate the transient state with an increased risk of periodontitis development. The genus *Capnocytophaga* is represented in samples from the transient state by three species: HMT 775 *Capnocytophaga sputigena* (2.63%), HMT329 *Capnocytophaga leadbetteri* (1.91%), and CT51 *Capnocytophaga granulosa* (1.15%). It is not a highly abundant genus but its relative abundance in healthy samples is remarkably lower and in periodontitis, it almost disappears. This finding is in good agreement with Pudakalkatti et al., who described *Capnocytophaga* species to be the most prevalent in gingivitis (a transient state from a clinical point of view) rather than in healthy periodontium and periodontitis (Pudakalkatti et al., 2016). They also claimed that *Capnocytophaga* has the potential to cause periodontal disease, but as it is less competitive in the periodontal pocket, it is usually overgrown by other rapidly growing bacteria. The role of *Capnocytophaga* is also supported by experiments published by Okuda et al., who proved that biofilm formation by *F. nucleatum* is enhanced by a soluble factor produced by *Capnocytophaga* cells (Okuda et al., 2012). Another taxon exhibiting the highest average relative abundance and prevalence in the transient group is HMT311 *Prevotella oris* (1.20%), a taxon previously proved to co-aggregate with *P. gingivalis* and thus to promote the colonization of the gingiva by *P. gingivalis* in early stage of biofilm formation (Sato and Nakazawa, 2014). Finally, CT53 *Tannerella* sp. and HMT623 *Campylobacter gracilis* were also associated with the transient state. CT53 is comprised of three *Tannerella* species, two of them (HMT808, and HMT916) associated with periodontitis (Griffen et al., 2012; Beall et al., 2018), while HMT286 having a relationship to oral health (Leys et al., 2002). The 16S rDNA region sequenced in this study does not allow us to differentiate these three species, thus preventing the meaningful discussion of their role in the development of periodontitis. The average relative abundance of HMT623 *C. gracilis* in periodontal health and periodontitis is comparable and almost negligible (<0.4%). In transient state, it increased above 1% and also the prevalence was remarkably higher (89% compared to 43% and 64% in both border states). This finding corresponds well with previous association of *C. gracilis* with shallow periodontal pockets rather than the deeper ones (Macuch and Tanner, 2000). As a microaerophilic organism, which requires an environment that contains a reduced concentration of oxygen (Guillermo et al., 1996), *C. gracilis* could be, together with the above mentioned transient state-associated taxa, another supporting indicator of the initiating dysbiosis and increased risk of periodontitis development.

### OM Changes in Aging

Belibasakis in his recent review summarized the knowledge concerning the OM composition changes in relation to the aging (Belibasakis, 2018) showing that relatively simple OM in early childhood is enriched by the acquisition of new taxa at an early predentate imprinting period and later during the eruption of primary teeth. During adult life, the OM composition of healthy individuals tends to keep a dynamically balanced state called “microbial homeostasis” comprising both natural and repeated colonization of the oral cavity by novel taxa without a remarkable effect on oral health. However, aging does result in changes to the host immune system, which in turn shifts the tolerance against microbial inhabitants of the oral cavity and which could consequently cause dysbiosis and periodontal disease. Besides the increasing prevalence of *Actinomyces* spp. in samples from older individuals (in spite of the increased prevalence of exposed root surfaces in higher age), no considerable differences in the OM composition were noted with regards to dental caries or periodontitis, between younger and elderly healthy populations (Belibasakis, 2018).

Our analysis, nevertheless, revealed several oral taxa clearly more abundant and/or prevalent in HO group when compared to the HY and opposite, even though the age difference between both groups of healthy individuals is not big (40-53 years with average 46 in HO group *vs.* 19-39 years with average 23 in HY). Generally, these changes could be summarized as an aging-related gradual decrease of relative abundance of health-associated taxa and an increase of taxa associated with the transient state. The most remarkable is the increasing relative abundance of CT3 *F. nucleatum* and *Capnocytophaga* species and decreasing relative abundance of health-associated *Neisseria* species, *Lautropia mirabilis*, *Prevotella histicola* or *Gemella morbillorum* (**Table 2**). Quite specific is a case of CT8 *P. pasteri*/*P. catoniae*, which average relative abundance clearly decreases with growing age (5.19% in HY *vs.* 1.59% in HO), but according to the **Table 1** it is a typical taxon for the transient state (2.15% in health vs. 13.05% in a transient state and only 0.37% in periodontitis). When considering solely the HY group, the average relative abundance of CT8 in 19 transient samples is 15.9% while in 72 remaining samples it is only 2.35%. Similarly, in 6 AP samples in the transient area, the average relative abundance of CT8 is 6.49% while in the remaining 39 AP samples it is only 0.37%. Members of CT8 *P. pasteri*/*P. catoniae* belong to the so-called POTG (*Porphyromonas* other than *gingivalis*) group of microorganisms (Guilloux et al., 2020). Typically, they colonize lungs and lower airways and in some diseases like cystic fibrosis, the relative abundance of *P. catoniae* can serve as a marker to discriminate between various states of health (Cuthbertson et al., 2016). POTG and mainly CT8 taxa were also frequently identified in the oral cavity, but in contrast to *P. gingivalis*, they were associated with periodontal health (Abusleme et al., 2013; Camelo-Castillo et al., 2015). Our data, however, indicate, that the increased relative abundance of CT8 is rather a marker of the transient state directing to periodontitis (see small panel in **Figure 3**).

The increased average relative abundance of periodontitis associated taxa in HO samples corresponds well with frequently reported higher prevalence and severity of periodontal disease among older adults (Eke et al., 2012; Baelum and López, 2013; Eke et al., 2015; Feres et al., 2016; Ebersole et al., 2018). Generally, the aging comes with risk factors like a higher predisposition to other systemic diseases which can indirectly modulate the periodontal condition (Persson, 2018), the excessive immune response of the host to oral microbiota (Ebersole et al., 2016) resulting in aging-related moderate loss of periodontal attachment and alveolar bone (Burt, 1994) or higher incidence of the exposed root surfaces, facilitating overgrowth of opportunistic pathogens. The age was described to be a significant factor driving the OM composition dynamics (Belibasakis, 2018; Deshpande et al., 2018), however, the causality still remains unclear (Feres et al., 2016; LaMonte et al., 2019).

### The Stomatotypes in Periodontal Health and Microbial Succession

The OM compositional patterns representing various global optimal equilibria of the microbial community have recently been referred to as “stomatotypes” (Willis et al., 2018; Willis and Gabaldón, 2020). The yet identified stomatotypes from systematically healthy individuals (De Filippis et al., 2014; Takeshita et al., 2016; Zaura et al., 2017; Willis et al., 2018) are summarized in **Table 3** together with stomatotypes identified in this study.

**Table 3:** OM stomatotypes in periodontal health.

Authors	Age of subjects	No. of subjects	Country	Sample type	Medical/dental examination	Stomatotype designation	The determining taxa		Method
**Current study**	**19-53**	**108**	**Czech Republic**	**Dental plaque from the gingival sulcus**	**All probands examinated by periodontologist: no periodontal pocket > 3mm**	**Cluster 1**	**CT2 *Streptococcus mitis/oralis*** **HMT718, CT23, and CT24 *Haemophilus* spp.**		**Illumina MiSeq**
						**Cluster 2**	**CT6 *Veillonella rogosae/dispar*** **CT25, CT27 *Neisseria*** **CT37 *Gemella morbillorum***		
						**Cluster 3a**	**CT43 *Streptococcus gordonii*** **CT48 *Rothia aeria***		
						**Cluster 3b**	**HMT14, CT27 *Neisseria* spp.** **CT33 Class Bacilli**		
Willis et al. (2018)	13-15	1319	Iberian Peninsula and Balearic Islands	Saliva- mouth wash	Without dental and medical exclusion criteria	stomatotype 1	*Neisseria, Haemophilus*		Illumina MiSeq
						stomatotype 2	*Veillonella, Prevotella*,	*Streptococcus*,	
Zaura et al. (2017)	18-32	268	The Netherlands	Unstimulated saliva	Systemically healthy individuals, sampling during a regular check-up at the dentist.	MIC3	*Veillonella atypica/Veillonella dispar and Prevotella*		Illumina MiSeq
						MIC2	*Streptococcus mitis group Streptococcus gordonii* *Rothia mucilaginosa*		
						MIC1.3	*Neisseria flavescens Neisseria subflava and Haemophilus parainfluenzae*		
						MIC1.2	*Streptococcus salivarius, Streptococcus vestibularis, Streptococcus australis Streptococcus parasanguinis* and *Granulicatella adiacens*		
						MIC1.1	*Prevotella* sp. HMT313 and *Paraprevotella/Alloprevotella* sp. HMT308		
De Filippis et al. (2014)	18–55	161	Italy	Unstimulated saliva	Systemically healthy individuals. No information about dental examination.	cluster I	*Neisseria, Fusobacterium, Porphyromonas*		454
						cluster II	*Prevotella*		
						cluster III	*Streptococcus, Gemella, Porphyromonas*		
Takeshita et al. (2016)	> 40	2343	Japan	Stimulated saliva	Dental and medical examinations were performed on 68.2% individuals	type I	*Veillonella, Prevotella, Actinomyces, Rothia, S. salivarius*, and *S. parasanguinis*		Ion PGM
						type II	*Streptococcus mitis, Haemophilus, Porphyromonas, Gemella*, and *Neisseiria*		

Mutually corresponding stomatotypes are color-coded. CT stands for combined taxon (See **Supplementary Table 3**).

The comparability of the data is slightly limited by inconsistent or even missing subject characterization and examination of periodontal health before sampling. Additionally, all the other OM samples were isolated from saliva, which differs in microbial composition from subgingival plaque. Certain variability in the taxonomic composition of the identified stomatotypes can also be given by demographic differences such as the drinking water source (Willis et al., 2018) or the prevailing diet (Lassalle et al., 2018). Nevertheless, the separate clustering of *Streptococcus* and *Veillonella* based OMs was generally observed in other studies as well (Takeshita et al., 2016; Zaura et al., 2017).

Considering the current knowledge of the microbial succession in the oral cavity during the onset of periodontal disease, and the characteristics of identified genera, we could hypothesize, that only the *Streptococcus*-based Cluster 1 represents healthy OM, while the Cluster 2 could already represent the initial dysbiotic state. The Cluster 1 stomatotype, and also the outliers Cluster 3a and Cluster 3b, are characterized by the predominant presence of early colonizers like *Streptococcus*, *Neisseria* (Mahajan et al., 2013), *Haemophilus* (Kolenbrander et al., 1993), and *Rothia* (Sulyanto et al., 2019) involved in the initial plaque formation. Samples from the stomatotype Cluster 2 also contain early colonizers of genera *Neisseria* and *Gemella* (Mahajan et al., 2013), but most remarkably they are exceptionally rich in *Veillonella* species. The genus *Veillonella* is considered to be a pioneer colonizer as well (Sulyanto et al., 2019), but among others, it is the only highly abundant anaerobic taxon assigned generally to periodontal health. *Veillonella* species possess two characteristics that rank them among the most important bridging taxa in the oral biofilm community. They can utilize the lactate generated mainly by streptococci as their primary energy and carbon source, and they produce catalase protecting *F. nucleatum* and other more fastidious anaerobes against hydrogen peroxide (Rogosa and Bishop, 1964). *Veillonella* also produces nutrients for the survival and growth of periodontal pathogens (Zhou et al., 2017). Therefore, we hypothesize, that the stomatotype Cluster 2 still represents clinically healthy individuals but already with an increased risk of periodontitis development. A further stage in the disease onset and progression could be represented by Cluster 4 (the transient state) with the increased relative abundance of anaerobic CT3 *F. nucleatum* and CT8 *Porphyromonas pasteri/catoniae* but still no or a negligible amount of true periopathogens and mostly no clinical signs of the disease. *F. nucleatum* forms a coaggregation bridge between early aerobic colonizers and other bacteria including anaerobic members of the red complex (*P. gingivalis*, *T. forsythia*, and *T. denticola*) (Bradshaw et al., 1998; Mahajan et al., 2013). This ability of *F. nucleatum* to coaggregate with a wide variety of partner strains is highly unusual (Kolenbrander et al., 2002; Kolenbrander et al., 2006). It has been shown that fusobacteria play a role in protecting against atmospheric oxygen and hydrogen peroxide in the oral biofilm and even support the growth of anaerobes, such as *Porphyromonas gingivalis*, under aerated conditions (Diaz et al., 2002). The presence of *F. nucleatum* in a higher amount thus would enable the periodontitis-associated bacteria to overgrowth the first colonizers.

Nevertheless, it is not only the taxonomic composition of the OM but the overall metabolic activity in the oral habitat including the host response to microbial production, which are the critical factors distinguishing between oral health and dysbiosis resulting in any type of oral pathogenesis. In the majority (~ 90%) of samples, the OM taxonomic composition corresponds well to the state of health and can serve as a fast diagnostic tool, however, still, there are individuals with atypical OM taxonomic composition, the atypical metabolic activity of typical OM or unusual immune reaction toward usual OM – in all these cases, the evaluation of proteome and/or metabolome could provide a more accurate image.

## Data Availability Statement

The datasets presented in this study can be found in online repositories. The names of the repository/repositories and accession number(s) can be found below: https://www.ncbi.nlm.nih.gov/bioproject, PRJNA670573.

## Ethics Statement

The studies involving human participants were reviewed and approved by Ethics Committee of the First Faculty of Medicine of Charles University and General University Hospital in Prague as a part of project No. 17-30753A of the Czech Health Research Council. The patients/participants provided their written informed consent to participate in this study.

## Author Contributions

JJ and LN postulated the hypothesis and designed the experiments. ML participated on experiment design, evaluated sequencing results and prepared first draft of the manuscript. JM diagnosed the patients and performed sampling. TJ assisted in sampling and performed DNA isolation and primary PCR. BT further processed samples for sequencing and participated on evaluation of sequencing results. OH did the clustering analysis and participated on evaluation of results and figure preparation. LN participated on the evaluation of results and finalized manuscript. All authors contributed to the article and approved the submitted version.

## Funding

This study was supported by grant 486417 from the Grant Agency of Charles University and project 17-30753A from Czech health research council.

## Conflict of Interest

The authors declare that the research was conducted in the absence of any commercial or financial relationships that could be construed as a potential conflict of interest.

## References

[B1] Abusleme L. Dupuy A. K. Dutzan N. Silva N. Burleson J. A. Strausbaugh L. D.. (2013). The subgingival microbiome in health and periodontitis and its relationship with community biomass and inflammation. Isme J. 7, 1016–1025. 10.1038/ismej.2012.174 23303375 PMC3635234

[B2] Armitage G. C. Cullinan M. P. (2010). Comparison of the clinical features of chronic and aggressive periodontitis. Periodontol. 2000 53, 12–27. 10.1111/j.1600-0757.2010.00353.x 20403102

[B3] Aronesty E. (2013). Comparison of sequencing utility programs. Open Bioinform. J. 7 (1), 1–8.

[B4] Baelum V. López R. (2013). Periodontal disease epidemiology - learned and unlearned? Periodontol. 2000 62, 37–58. 10.1111/j.1600-0757.2012.00449.x 23574463

[B5] Baldrian P. Kolaiřík M. Štursová M. Kopecký J. Valášková V. Větrovský T.. (2012). Active and total microbial communities in forest soil are largely different and highly stratified during decomposition. ISME J. 6, 248–258. 10.1038/ismej.2011.95 21776033 PMC3260513

[B6] Beall C. J. Campbell A. G. Griffen A. L. Podar M. Leys E. J. (2018). Genomics of the uncultivated, periodontitis-associated bacterium Tannerella sp. BU045 (Oral Taxon 808). mSystems 3 (3), e00018–18. 10.1128/msystems.00018-18 29896567 PMC5989130

[B7] Belibasakis G. N. (2018). Microbiological changes of the ageing oral cavity. Arch. Oral. Biol. 96, 230–232. 10.1016/j.archoralbio.2018.10.001 30308473

[B8] Bradshaw D. J. Marsh P. D. Watson K. G. Allison C. (1998). Role of Fusobacterium nucleatum and coaggregation in anaerobe survival in planktonic and biofilm oral microbial communities during aeration. Infect. Immun. 66, 4729–4732. 10.1128/iai.66.10.4729-4732.1998 9746571 PMC108582

[B9] Burt B. A. (1994). Periodontitis and aging: reviewing recent evidence. J. Am. Dent Assoc. 125, 273–279. 10.14219/jada.archive.1994.0034 8157839

[B10] Camelo-Castillo A. J. Mira A. Pico A. Nibali L. Henderson B. Donos N.. (2015). Subgingival microbiota in health compared to periodontitis and the influence of smoking. Front. Microbiol. 6, 119. 10.3389/fmicb.2015.00119 25814980 PMC4356944

[B11] Caton J. G. Armitage G. Berglundh T. Chapple I. L. Jepsen S. Kornman K. S.. (2018). A new classification scheme for periodontal and peri-implant diseases and conditions–Introduction and key changes from the 1999 classification. J. Periodontol 89, S1–S8. 10.1002/JPER.18-0157 29926946

[B12] Chen T. Yu W. H. Izard J. Baranova O. V. Lakshmanan A. Dewhirst F. E. (2010). The Human Oral Microbiome Database: a web accessible resource for investigating oral microbe taxonomic and genomic information. Database 2010. 10.1093/database/baq013 PMC2911848 20624719

[B13] Cuthbertson L. Rogers G. B. Walker A. W. Oliver A. Green L. E. Daniels T. W. V.. (2016). Respiratory microbiota resistance and resilience to pulmonary exacerbation and subsequent antimicrobial intervention. ISME J. 10, 1081–1091. 10.1038/ismej.2015.198 26555248 PMC4820042

[B14] De Filippis F. Vannini L. La Storia A. Laghi L. Piombino P. Stellato G.. (2014). The same microbiota and a potentially discriminant metabolome in the saliva of omnivore, ovo-lacto-vegetarian and vegan individuals. PloS One 9 (11), e112373. 10.1371/journal.pone.0112373 25372853 PMC4221475

[B15] De Lillo A. Booth V. Kyriacou L. Weightman A. J. Wade W. G. (2004). Culture-independent identification of periodontitis-associated Porphyromonas and Tannerella populations by targeted molecular analysis. J. Clin. Microbiol. 42, 5523–5527. 10.1128/JCM.42.12.5523-5527.2004 15583276 PMC535285

[B16] Deo P. N. Deshmukh R. (2019). Oral microbiome: Unveiling the fundamentals. J. Oral. Maxillofac Pathol. JOMFP 23, 122–128. 10.4103/jomfp.JOMFP PMC6503789 31110428

[B17] Deshpande N. P. Riordan S. M. Castaño-Rodríguez N. Wilkins M. R. Kaakoush N. O. (2018). Signatures within the esophageal microbiome are associated with host genetics, age, and disease. Microbiome 6, 1–14. 10.1186/s40168-018-0611-4 30558669 PMC6297961

[B18] Diaz P. I. Zilm P. S. Rogers A. H. (2002). Fusobacterium nucleatum supports the growth of Porphyromonas gingivalis in oxygenated and carbon-dioxide-depleted environments. Microbiology 148, 467–472. 10.1099/00221287-148-2-467 11832510

[B19] Dioguardi M. Alovisi M. Crincoli V. Aiuto R. Malagnino G. Quarta C.. (2020). Prevalence of the genus Propionibacterium in primary and persistent endodontic lesions: A systematic review. J. Clin. Med. 9 (3), 739. 10.3390/jcm9030739 PMC7141369 32182900

[B20] Dowd S. E. Callaway T. R. Wolcott R. D. Sun Y. Mckeehan T. Hagevoort R. G.. (2008). Evaluation of the bacterial diversity in the feces of cattle using 16S rDNA bacterial tag-encoded FLX amplicon pyrosequencing (bTEFAP). BMC Microbiol. 8. 10.1186/1471-2180-8-125 PMC2515157 18652685

[B21] Duran-Pinedo A. E. Frias-Lopez J. (2015). Beyond microbial community composition: functional activities of the oral microbiome in health and disease. Microbes Infect. 17, 505–516. 10.1016/j.micinf.2015.03.014 25862077 PMC4495649

[B22] Ebersole J. L. Graves C. L. Gonzalez O. A. Dawson D. Morford L. A. Huja P. E.. (2016). Aging, inflammation, immunity and periodontal disease. Periodontol. 2000 72, 54–75. 10.1111/prd.12135 27501491

[B23] Ebersole J. L. Al-Sabbagh M. Gonzalez O. A. Dawson D. R. (2018). Ageing effects on humoral immune responses in chronic periodontitis. J. Clin. Periodontol. 45, 680–692. 10.1111/jcpe.12881 29476652 PMC5992058

[B24] Edgar R. C. (2010). Search and clustering orders of magnitude faster than BLAST. Bioinformatics 26, 2460–2461. 10.1093/bioinformatics/btq461 20709691

[B25] Eke P. I. Dye B. A. Wei L. Thornton-Evans G. O. Genco R. J. (2012). Prevalence of periodontitis in adults in the United States: 2009 and 2010. J. Dent Res. 91, 914–920. 10.1177/0022034512457373 22935673

[B26] Eke P. I. Dye B. A. Wei L. Slade G. D. Thornton-Evans G. O. Borgnakke W. S.. (2015). Update on prevalence of periodontitis in adults in the United States: NHANES 2009 – 2012. J. Periodontol. 86, 611–622. 10.1016/j.physbeh.2017.03.040 25688694 PMC4460825

[B27] Eke P. I. Wei L. Borgnakke W. S. Thornton-Evans G. Zhang X. Lu H.. (2016). Periodontitis prevalence in adults ≥ 65 years of age, in the USA. Periodontol. 2000 72, 76–95. 10.1111/prd.12145 27501492 PMC8223257

[B28] Feres M. Teles F. Teles R. Figueiredo L. C. Faveri M. (2016). The subgingival periodontal microbiota of the aging mouth. Periodontol. 2000 72, 30–53. 10.1111/prd.12136.The 27501490 PMC5141605

[B29] Frencken J. E. Sharma P. Stenhouse L. Green D. Laverty D. Dietrich T. (2017). Global epidemiology of dental caries and severe periodontitis – a comprehensive review. J. Clin. Periodontol. 44, S94–S105. 10.1111/jcpe.12677 28266116

[B30] Griffen A. L. Beall C. J. Campbell J. H. Firestone N. D. Kumar P. S. Yang Z. K.. (2012). Distinct and complex bacterial profiles in human periodontitis and health revealed by 16S pyrosequencing. ISME J. 6, 1176–1185. 10.1038/ismej.2011.191 22170420 PMC3358035

[B31] Guillermo I. Perez-Perez Blaser M. J. (1996). “Chapter 23 Campylobacter and Helicobacter,” In: Baron S. Medical Microbiology, 4th edition (University of Texas Medical Branch at Galveston). 21413331

[B32] Guilloux C. Lamoureux C. Beauruelle C. Héry-Arnaud G. (2020). Porphyromonas: A neglected potential key genus in human microbiomes. Anaerobe. 10.1016/j.anaerobe.2020.102230 32615270

[B33] Hagberg A. Swart P. Chult D. (2008). “Exploring network structure, dynamics, and function using NetworkX. @,” in Proceedings of the 7th Python in Science Conference. Eds. Gäel Varoquaux J. M. Vaught T., 11–15.

[B34] Hajishengallis G. (2015). Periodontitis: from microbial immune subversion to systemic inflammation. Nat. Rev. Immunol. 15, 30–44. 10.1038/nri3785 25534621 PMC4276050

[B35] Hammer Ø. Harper D. A. T. Ryan P. D. (2001). Past: Paleontological statistics software package for education and data analysis. Palaeontol Electron. 4.1.4, 1–9.

[B36] He J. Huang W. Pan Z. Cui H. Qi G. Zhou X.. (2012). Quantitative analysis of microbiota in saliva, supragingival, and subgingival plaque of Chinese adults with chronic periodontitis. Clin. Oral. Investig. 16, 1579–1588. 10.1007/s00784-011-0654-4 22169888

[B37] Henderson B. Ward J. M. Ready D. (2010). Aggregatibacter (Actinobacillus) actinomycetemcomitans: A triple A* periodontopathogen? Periodontol. 2000 54, 78–105. 10.1111/j.1600-0757.2009.00331.x 20712635

[B38] Hernández M. Dutzan N. García-Sesnich J. Abusleme L. Dezerega A. Silva N.. (2011). Host-pathogen interactions in progressive chronic periodontitis. J. Dent Res. 90, 1164–1170. 10.1177/0022034511401405 21471325

[B39] Kirst M. E. Li E. C. Alfant B. Chi Y. Y. Walker C. Magnusson I.. (2015). Dysbiosis and alterations in predicted functions of the subgingival microbiome in chronic periodontitis. Appl. Environ. Microbiol. 81, 783–793. 10.1128/AEM.02712-14 25398868 PMC4277562

[B40] Kolenbrander P. E. Ganeshkumar N. Cassels F. J. Hughes C. V. (1993). Coaggregation: specific adherence among human oral plaque bacteria. FASEB J. 7, 406–413. 10.1096/fasebj.7.5.8462782 8462782

[B41] Kolenbrander P. E. Andersen R. N. Blehert D. S. Egland P. G. Foster J. S. Palmer R. J. (2002). Communication among oral bacteria. Microbiol. Mol. Biol. Rev. 66, 486–505. 10.1128/mmbr.66.3.486-505.2002 12209001 PMC120797

[B42] Kolenbrander P. E. Palmer R. J. Rickard A. H. Jakubovics N. S. Chalmers N. I. Diaz P. I. (2006). Bacterial interactions and successions during plaque development. Periodontol. 2000 42, 47–79. 10.1111/j.1600-0757.2006.00187.x 16930306

[B43] Kolenbrander P. E. Palmer R. J. Periasamy S. Jakubovics N. S. (2010). Oral multispecies biofilm development and the key role of cell-cell distance. Nat. Rev. Microbiol. 8, 471–480. 10.1038/nrmicro2381 20514044

[B44] LaMonte M. J. Genco R. J. Buck M. J. McSkimming D. I. Li L. Hovey K. M.. (2019). Composition and diversity of the subgingival microbiome and its relationship with age in postmenopausal women: An epidemiologic investigation. BMC Oral. Health 19, 246. 10.1186/s12903-019-0906-2 31722703 PMC6854792

[B45] Lane D. J. Pace B. Olsen G. J. Stahlt D. A. Sogin M. L. Pace N. R. (1985). Rapid determination of 16S ribosomal RNA sequences for phylogenetic analyses. Proc. Natl. Acad. Sci. U S A 82, 6955–6959. 10.1073/pnas.82.20.6955 2413450 PMC391288

[B46] Lassalle F. Spagnoletti M. Fumagalli M. Shaw L. Dyble M. Walker C.. (2018). Oral microbiomes from hunter-gatherers and traditional farmers reveal shifts in commensal balance and pathogen load linked to diet. Mol. Ecol. 27, 182–195. 10.1111/mec.14435 29165844

[B47] Leys E. J. Lyons S. R. Moeschberger M. L. Rumpf R. W. Griffen A. L. (2002). Association of Bacteroides forsythus and a novel Bacteroides phylotype with periodontitis. J. Clin. Microbiol. 40, 821–825. 10.1128/JCM.40.3.821-825.2002 11880400 PMC120258

[B48] Liu B. Faller L. L. Klitgord N. Mazumdar V. Ghodsi M. Sommer D. D.. (2012). Deep sequencing of the oral microbiome reveals signatures of periodontal disease. PloS One 7 (6), e37919. 10.1371/journal.pone.0037919 22675498 PMC3366996

[B49] López R. Smith P. Göstemeyer G. Schwendicke F. (2017). Ageing, dental caries and periodontal diseases. J. Clin. Periodontol. 44, S145–S152. 10.1111/jcpe.12683 28266118

[B50] Macuch P. J. Tanner A. C. (2000). Campylobacter species in health, gingivitis, and periodontitis. J. Dent Res. 79, 785–792. 10.1177/00220345000790021301 10728981

[B51] Mahajan A. Singh B. Kashyap D. Kumar A. Mahajan P. (2013). Interspecies communication and periodontal disease. Sci. World J. 2013. 10.1155/2013/765434 PMC3874309 24396307

[B52] Meuric V. Gall-David S. Boyer E. Acuña-Amador L. Martin B. Fong S. B.. (2017). Signature of microbial dysbiosis in periodontitis. Appl. Environ. Microbiol. 83, e00462–17. 10.1128/AEM.00462-17 28476771 PMC5494626

[B53] Monnet-Corti V. Antezack A. Moll V. (2018). Vestibular frenectomy in periodontal plastic surgery. J. Dentofac Anomalies Orthod. 21 (2), 205. 10.1051/odfen/2018054

[B54] Najmanova L. Sabova L. Lenartova M. Janatova T. Mysak J. Vetrovsky T.. (2021). R/G value – a numeric index of periodontal health. Front. Cell. Inf. Microbiol 11, 602643. 10.3389/fcimb.2021.602643 PMC7988090 33777830

[B55] Okuda T. Okuda K. Kokubu E. Kawana T. Saito A. Ishihara K. (2012). Synergistic effect on biofilm formation between Fusobacterium nucleatum and Capnocytophaga ochracea. Anaerobe 18, 157–161. 10.1016/j.anaerobe.2012.01.001 22252100

[B56] Park O.-J. Yi H. Jeon J. H. Kang S.-S. Koo K.-T. Kum K.-Y.. (2015). Pyrosequencing analysis of subgingival microbiota in distinct periodontal conditions. J. Dent Res. 94, 921–927. 10.1177/0022034515583531 25904141

[B57] Pérez-Chaparro P. Gonçalves C. Figueiredo L. Faveri M. Lobão E. Tamashiro N.. (2014). Newly identified pathogens associated with periodontitis: a systematic review. J. Dent Res. 93, 846–858. 10.1177/0022034514542468 25074492 PMC4541103

[B58] Persson G. R. (2018). Periodontal complications with age. Periodontol. 2000 78, 185–194. 10.1111/prd.12227 30198125

[B59] Pudakalkatti P. S. Baheti A. S. Hattarki S. A. Kambali S. S. Naik R. M. (2016). Detection and prevalence of Capnocytophaga in periodontal health and disease. J. Orofac Sci. 8, 92–95. 10.4103/0975-8844.195911

[B60] Rogosa M. Bishop F. S. (1964). The genus Veillonella. J. Bacteriol. 88, 37–41. 10.1128/jb.88.1.37-41.1964 14198791 PMC277253

[B61] Rossum G. V. Drake F. The Python Language Reference. Amsterdam, Netherlands: Python Software Foundation (2010).

[B62] Rusthen S. Kristoffersen A. K. Young A. Galtung H. K. Petrovski B. É. Palm Ø.. (2019). Dysbiotic salivary microbiota in dry mouth and primary Sjögren’s syndrome patients. PloS One 14 (6), e0218319. 10.1371/journal.pone.0218319 31211815 PMC6581286

[B63] Sato T. Nakazawa F. (2014). Coaggregation between Prevotella oris and Porphyromonas gingivalis. J. Microbiol. Immunol. Infect. 47, 182–186. 10.1016/j.jmii.2012.09.005 23245806

[B64] Schacher B. Baron F. Roßberg M. Wohlfeil M. Arndt R. Eickholz P. (2007). Aggregatibacter actinomycetemcomitans as indicator for aggressive periodontitis by two analysing strategies. J. Clin. Periodontol. 34, 566–573. 10.1111/j.1600-051X.2007.01080.x 17433043

[B65] Socransky S. S. Haffajee A. D. Cugini M. A. Smith C. Kent R. L. (1998). Microbial complexes in subgingival plaque. J. Clin. Periodontol. 25, 134–144. 10.1111/j.1600-051X.1998.tb02419.x 9495612

[B66] Stoddard S. F. Smith B. J. Hein R. Roller B. R. K. Schmidt T. M. (2015). rrnDB: Improved tools for interpreting rRNA gene abundance in bacteria and archaea and a new foundation for future development. Nucleic Acids Res. 43, D593–D598. 10.1093/nar/gku1201 25414355 PMC4383981

[B67] Sultan A. S. Kong E. F. Rizk A. M. Jabra-Rizk M. A. (2018). The oral microbiome: A Lesson in coexistence. PloS Pathog. 14, e1006719. 10.1371/journal.ppat.1006719 29370304 PMC5784999

[B68] Sulyanto R. M. Thompson Z. A. Beall C. J. Leys E. J. Griffen A. L. (2019). The Predominant Oral Microbiota Is Acquired Early in an Organized Pattern. Sci. Rep. 9, 1–8. 10.1038/s41598-019-46923-0 31332213 PMC6646312

[B69] Szafranski S. P. Wos-Oxley M. L. Vilchez-Vargas R. Jáuregui R. Plumeier I. Klawonn F.. (2015). High-resolution taxonomic profiling of the subgingival microbiome for biomarker discovery and periodontitis diagnosis. Appl. Environ. Microbiol. 81, 1047–1058. 10.1128/AEM.03534-14 25452281 PMC4292489

[B70] Takeshita T. Kageyama S. Furuta M. Tsuboi H. Takeuchi K. Shibata Y.. (2016). Bacterial diversity in saliva and oral health-related conditions: the Hisayama Study. Sci. Rep. 6, 22164. 10.1038/srep22164 26907866 PMC4764907

[B71] Tsai C. Y. Tang C. Y. Tan T. S. Chen K. H. Liao K. H. Liou M. L. (2018). Subgingival microbiota in individuals with severe chronic periodontitis. J. Microbiol. Immunol. Infect. 51, 226–234. 10.1016/j.jmii.2016.04.007 27262209

[B72] Van der Velden U. (2017). What exactly distinguishes aggressive from chronic periodontitis: is it mainly a difference in the degree of bacterial invasiveness? Periodontol. 2000 75, 24–44. 10.1111/prd.12202 28758297

[B73] Větrovský T. Baldrian P. Morais D. (2018). SEED 2: A user-friendly platform for amplicon high-throughput sequencing data analyses. Bioinformatics 34, 2292–2294. 10.1093/bioinformatics/bty071 29452334 PMC6022770

[B74] Větrovský T. Baldrian P. (2013). The variability of the 16S rRNA gene in bacterial genomes and its consequences for bacterial community analyses. PloS One 8. 10.1371/journal.pone.0057923 PMC3583900 23460914

[B75] Willis J. R. González-Torres P. Pittis A. A. Bejarano L. A. Cozzuto L. Andreu-Somavilla N.. (2018). Citizen science charts two major “stomatotypes” in the oral microbiome of adolescents and reveals links with habits and drinking water composition. Microbiome 6, 218. 10.1186/s40168-018-0592-3 30522523 PMC6284318

[B76] Willis J. R. Gabaldón T. (2020). The human oral microbiome in health and disease: From sequences to ecosystems. Microorganisms 8, 308. 10.3390/microorganisms8020308 PMC7074908 32102216

[B77] Yang N. Y. Zhang Q. Li J. L. Yang S. H. Shi Q. (2014). Progression of periodontal inflammation in adolescents is associated with increased number of Porphyromonas gingivalis, Prevotella intermedia, Tannerella forsythensis, and Fusobacterium nucleatum. Int. J. Paediatr. Dent 24, 226–233. 10.1111/ipd.12065 24025042

[B78] Yasunaga H. Takeshita T. Shibata Y. Furuta M. Shimazaki Y. Akifusa S.. (2017). Exploration of bacterial species associated with the salivary microbiome of individuals with a low susceptibility to dental caries. Clin. Oral. Investig. 21, 2399–2406. 10.1007/s00784-016-2035-5 28013437

[B79] Zaura E. Brandt B. W. Prodan A. Teixeira De Mattos M. J. Imangaliyev S. Kool J.. (2017). On the ecosystemic network of saliva in healthy young adults. ISME J. 11, 1218–1231. 10.1038/ismej.2016.199 28072421 PMC5475835

[B80] Zhou P. Li X. Huang I.-H. Qi F. (2017). Veillonella catalase protects the growth of Fusobacterium nucleatum in microaerophilic and Streptococcus gordonii-resident environments. Appl. Environ. Microbiol. 83, e01079–17. 10.1128/AEM.01079-17 28778894 PMC5601340

